# Magnesium Levels and Diastolic Blood Pressure (DBP) as a Vasospasm Prediction Metric in Patients With Aneurysmal Subarachnoid Hemorrhage (SAH)

**DOI:** 10.7759/cureus.23161

**Published:** 2022-03-14

**Authors:** Paras Savla, Maxwell A Marino, Dario A Marotta, James Brazdzionis, Saman Farr, Stacey Podkovik, James Wiginton, Emilio C Tayag, Vladimir Cortez, Dan E Miulli

**Affiliations:** 1 Neurosurgery, Riverside University Health System Medical Center, Moreno Valley, USA; 2 Department of Research, Alabama College of Osteopathic Medicine, Dothan, USA; 3 Department of Neurology, Division of Neuropsychology, University of Alabama, Birmingham, USA; 4 Neurology and Neurosurgery, Desert Regional Medical Center, Palm Springs, USA; 5 Neurosurgery, Desert Regional Medical Center, Palm Springs, USA; 6 Neurosurgery, Arrowhead Regional Medical Center, Colton, USA

**Keywords:** neuro-critical care, diastolic blood pressure, magnesium, vasospasm, subarachnoid hemorrhage

## Abstract

Introduction

Vasospasm is a significant cause of morbidity and mortality in patients with aneurysmal subarachnoid hemorrhage (SAH). The purpose of this study is to evaluate a possible link between vasospasm in patients with aneurysmal SAH and magnesium and blood pressure levels.

Methods

Subjects were selected based on chart review of patients presenting to a comprehensive stroke center in Southern California with aneurysmal SAH. 27 were included based on the following criteria: patients greater than 18 years of age, aneurysmal SAH, clinically symptomatic vasospasms and at least one diagnostic confirmation - either from a transcranial doppler (TCD) or digital subtraction angiogram (DSA). The following exclusion criteria also applied: 1) incomplete documentation in the medical record; 2) patients <18 years of age; and 3) patients without TCD measurements.

Results

In an overall analysis of all patients with or without vasospasm, it was found that the presence of vasospasm was significantly correlated with diastolic blood pressures (DBPs) on day of vasospasm with an r value of 0.418 and p<0.001. Average daily DBPs throughout hospital stay were also correlated with vasospasm with an r-value of 0.455 and p<0.001. Changes in magnesium overall were also significantly related to left Lindegaard ratios with an r value of -0.201 and p value of 0.032. Lindegaard ratios were significantly correlated with age with r values of 0.510, p<0.001, and r=-0.482, p<0.001 for left and right, respectively. A change in magnesium was inversely correlated to the left Lindegaard ratio with an n of 31 and p value of 0.014 (r= -0.439) in patients with vasospasm. We also found a lower incidence of vasospasm in patients older than 65.

Conclusion

Monitoring magnesium and increases in DBP might be effective as a prophylactic adjunct method in patients with SAH in an effort to predict clinical vasospasm.

## Introduction

Vasospasm is a significant cause of morbidity and mortality in patients with aneurysmal subarachnoid hemorrhage (SAH). Up to 40% of patients with SAH will develop vasospasm at some point in their care [1]. Predicting when a patient is in vasospasm is vital to the care of these patients, especially when clinical signs of vasospasm are not apparent. In order to determine whether a patient is in vasospasm, cerebral angiography and transcranial dopplers (TCDs) have traditionally been used to supplement the clinical exam [2]. However, the use of these technologies relies on availability of both a skilled operator and functioning instrumentation. In many clinical settings, one or more of those may not be readily available for rapid diagnosis. A surrogate determinant would be valuable in these cases.

Magnesium therapy in patients with aneurysmal SAH has been a very controversial topic. Studies have found variable outcomes from giving regular doses of magnesium sulfate to patients, due to a possible vasodilator effect decreasing the incidence of delayed cerebral ischemia that is caused by vasospasm [3,4]. Another study showed that magnesium could be as effective as nimodipine in reducing the incidence of delayed neurological deficits [5]. One major trial showed, however, that regular magnesium infusion did not provide clinical benefit [6]. However, the predictive effects of the temporal association of magnesium levels have not been well studied. The link between magnesium levels and the incidence and severity of vasospasm has not been evaluated thoroughly. The timing of which phenomenon occurs first has not been established. The body, in response to vasospasm, may be consuming high levels of magnesium for its vasodilatory effect, which could lead to low serum levels of magnesium [7]. However, it is possible that magnesium levels are low due to a different cause in SAH patients. This lower concentration of magnesium has less participation in vasodilatory affects possibly leading to higher chances of vasospasms.

The purpose of this study is to evaluate a possible link between vasospasm in patients with aneurysmal SAH and magnesium and blood pressure levels. Our hypothesis is that patients with low magnesium levels and high diastolic blood pressures (DBPs) will be in vasospasm, as confirmed by TCD measurements.

## Materials and methods

Study Design 

Subjects were selected based on chart review of patients presenting to a comprehensive stroke center in Southern California with aneurysmal SAH. Patient charts were selected based on ICD-10 code for aneurysmal SAH and reviewed for data.

Inclusion and Exclusion Criteria

Inclusion criteria were: patients greater than 18 years of age, aneurysmal SAH, clinically symptomatic vasospasms, and at least one diagnostic confirmation - either from a TCD or digital subtraction angiogram (DSA). The following exclusion criteria also applied: 1) incomplete documentation in the medical record; 2) patients <18 years of age; and 3) patients without TCD measurements.

Data Collection

A sample size of 37 was identified with aneurysmal SAH. Of the 37, 27 were included based on the inclusion and exclusion criteria. All patients received the institutional treatments of hypervolemia, 2 g of magnesium treatment twice per day, statin, nimodipine, and medication to keep systolic blood pressure (SBP) less than 130 prior to surgery and permissive hypertension after the aneurysm was secured. The data included age, gender, aneurysm location, daily magnesium levels, daily TCD and/or DSA values, daily vital signs including SBP and DBP, length of hospital and intensive care unit stay, and outcome at discharge.

Statistical Analysis

Statistical analysis was performed in SAS software for Windows. All statistical analyses were two-sided, and a p-value of <0.05 was considered statistically significant using Pearson coefficients.

## Results

A total of 37 patients were screened and identified with SAH on presentation to the medical center. 10 patients were excluded: seven patients were transferred to other facilities before any data was collected, and two expired, while one other patient did not undergo clinical testing for vasospasm. Therefore, there were 27 patients followed with SAH that had at least one diagnostic test demonstrating evidence offer evidence of vasospasm. A total of 126 data points were obtained from these patients.

Patients’ age ranged from 24 to 82 years old with a median age of 53. There were 17 males and 10 females. A total of 33.3% (9/27) of qualifying patients had evidence of at least one instance of vasospasm determined by either TCD or DSA. The distribution of vasospasms based on demographic factors is demonstrated in Table 1. This was broken down to 35.2% (6/17) of males and 30% (3/9) of females experienced a vasospasm at least one time throughout hospital stay.

**Table 1:** Patient demographics.

N = 27		
Sex	W/ Vasospasm	W/o Vasospasm
Male	6	11
Female	3	7
Total	9	18
Age		
20-29	1	1
30-39	3	1
40-49	1	3
50-59	2	3
60-69	1	4
70-79	1	4
80-89	0	2
Total	9	18

The correlations between all the factors obtained were assessed. It was found that the presence of vasospasm was significantly correlated with DBP on the day of vasospasm with an r value of 0.418 and p<0.001. Further, average daily DBPs throughout hospital stay were also correlated with vasospasm with an r-value of 0.455 and p<0.001. Changes in magnesium overall were also significantly related to left Lindegaard ratios with an r value of -0.201 and p value of 0.032. Lindegaard ratios were significantly correlated with age with r values of 0.510, p<0.001 and r=-0.482, p<0.001 for left and right, respectively.

Although not identified as correlated within all patients, when doing a subgroup analysis investigating vasospasm demonstrated through TCDs, it was found that a change in magnesium was inversely correlated to the left Lindegaard ratio with an n of 31 and p value of 0.014 (r=-0.439). A similar relationship was not seen in patients without vasospasm as there was no relationship between changes in magnesium and Lindegaard ratios (right or left) with p values of 0.129 and 0.623, respectively.

When utilizing t-testing to determine if there was a difference between the vasospasm and no-vasospasm groups, it was found that the DBP on day of vasospasm, average daily DBP, and DBP prior to the vasospasm event were significant, all with p<0.001.

**Figure 1:**
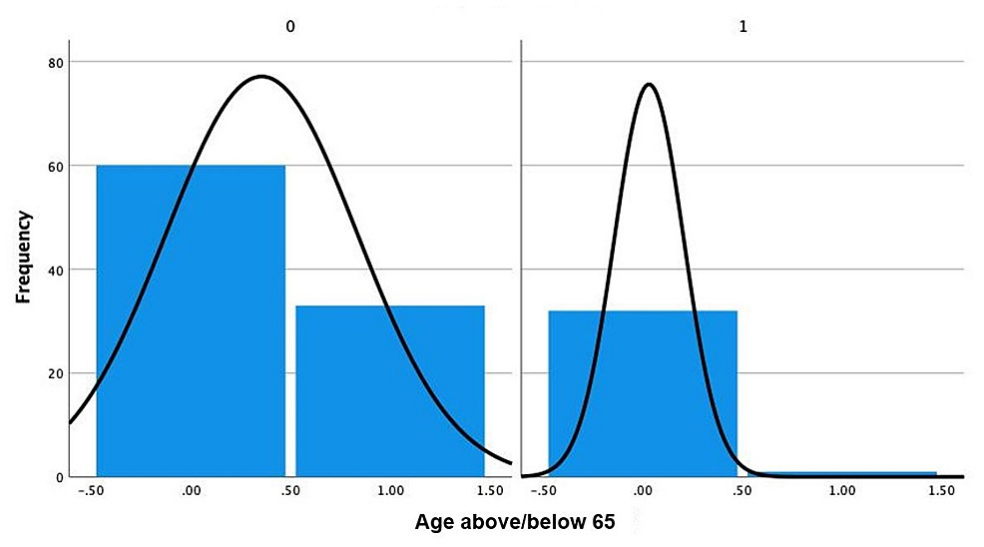
Histogram of patients with and without vasospasm, divided into two categories of age below and above 65. 0 indicates no vasospasm and 1 indicates vasospasm. The left bar in each chart indicates patients below the age of 65 and the right indicates patients above the age of 65.

We also found a lower incidence of vasospasm in patients older than 65. These data are highlighted in Figure 1.

## Discussion

The results of this retrospective analysis of magnesium levels in a population of patients with SAH carries several implications regarding the monitoring and the prospect of identifying vasospasm. Although prior studies have mixed results in the effectiveness of magnesium as a treatment for vasospasm [5-7], a temporal association used to predict vasospasm is revealed by our results. While patients who did not experience clinical vasospasm showed no relationship when magnesium levels trended downwards, these results demonstrate that patients who do experience vasospasm had a significant moderate relationship in decreasing magnesium levels compared with periods of active vasospasm. These findings demonstrate an adjunct clinical lab test that can help with diagnosis of cerebral vasospasms, potentially with a temporal predictive capability. This is especially important in patients who have already demonstrated vasospasm, as we can use additional lab values to monitor recurrence of vasospasm. Multiple studies evaluating high-dose magnesium as a prophylactic supplement to reduce the risk of poor outcomes in SAH has been explored, and with close monitoring of magnesium levels may warrant further investigation of developing protocols and guidelines for its routine dosing in patients of perceivably higher risk [8,9].

Our study did not demonstrate any significant association between those with vasospasm and SBP values. However, we did demonstrate the link between patients with vasospasm and elevated daily DBPs. Similarly, a retrospective analysis by Faust et al. demonstrated that increases in mean arterial pressure attributed to changes DBP were significant predictors of vasospasm and poor outcome [10]. With a larger sample size and further studies, a more direct link can be investigated and its use in treating patients with vasospasm can be broadened.

We also note a decrease in incidence of vasospasm in patients above the age of 65, as seen in Figure 1. Although prior studies have shown that older age may either result in increased vasospasm or have no link to vasospasm rate [3], our study shows a decrease in incidence. We postulate that older patients have more atherosclerotic vessels, so these vessels would be stiffer. However, this would need to be further investigated with a detailed analysis of the changes in stiffness and size of patients via angiography. Future studies could focus on this aspect to determine whether there truly is a link between stiffer vessels due to age and atherosclerosis and decreased incidence of vasospasm.

Limitations of this analytical method, as with all Pearson coefficient analysis, is that the linear relationship is assumed - decreasing magnesium levels lead to a linear increase in the risk of vasospasm. It is possible that the relationship may be nonlinear as well. Additionally, our results only demonstrate associations and not causation. There may be additional confounding variables within the pathophysiology of vasospasms that contribute to the findings that are encountered. It is well known that magnesium levels also affect calcium levels with a possible role for parathyroid hormone as well [11]. SAH is also a complicated pathologic process requiring close hemodynamic management as well as implications for controlling body temperature, coagulopathies, and blood sugar levels [12]. As a result, there are many confounding variables worth exploring that could yet still dwarf the significance of magnesium levels in the incidence of vasospasm. Future direction would be to prospectively correlate the more frequent temporal association between serum magnesium levels, with or without supplementation. There also needs to be a prospective study to correlate more frequent changes in DBP and the occurrence of vasospasms. This can then lead to the development of a predictive and readily available screening test. Also, as stated earlier, a larger patient population needs to be studied to make even more distinct connections and allow us to improve the treatment of patients with vasospasm. In facilities that do not have TCD and do not want to subject patients to daily angiograms, tracking lower blood concentrations of magnesium when given as twice daily supplementation or tracking the DBP may guide the neurointensivist towards the detection of continued occurrence of vasospasms.

## Conclusions

Monitoring magnesium might be effective as a prophylactic adjunct method in patients with SAH to predict clinical vasospasm. The moderate negative correlation demonstrates that a decrease in magnesium levels despite twice per day supplementation, at least in combination with signs and symptoms of vasospasm, is associated with a higher severity of clinical vasospasm. In addition, patients in vasospasm had a correlation to higher DBP values. As always, patients should be carefully observed in an intensive care setting with closed arterial blood pressure and ECG monitoring, as well as frequent measurements of magnesium levels, coagulation studies, blood sugar, and other routine labs and imaging as indicated.
